# Single-port nipple-sparing subcutaneous mastectomy with immediate prosthetic breast reconstruction for breast cancer

**DOI:** 10.1007/s00464-023-09862-6

**Published:** 2023-01-25

**Authors:** Zi-Han Wang, Guo-Xuan Gao, Wei-Hua Liu, Shan-Shan Wu, Fang Xie, Wei Xu, Guo-qian Ding, Ya-qian Xu, Zhong-tao Zhang, Xiang Qu

**Affiliations:** 1 Department of Breast Disease, Peking University People’s Hospital, Beijing, 100044 China; 2 Department of General Surgery, Beijing Friendship Hospital, Capital Medical University, 95 Yong-an Road, Xi-Cheng District, Beijing, 100050 China; 3 Surgery Department, Huairou Maternal and Child Health Care Hospital, Beijing Obstetrics and Gynecology Hospital, Capital Medical University, Beijing, 101400 China; 4 Department of Clinical Epidemiology and Evidence-Based Medicine, Beijing Friendship Hospital, 95 Yong-an Road, Xi-Cheng District, Beijing, 100050 China; 5 Breast Surgery, Beijing Hospital of Traditional Chinese Medicine Affiliated to Capital Medical University, Beijing, 100010 China

**Keywords:** Breast neoplasms, Endoscopic surgery, Prosthetic implantation, Breast reconstruction, Postoperative complication

## Abstract

**Introduction:**

This study compares the perioperative results, aesthetic outcome and oncologic safety of single-port insufflation endoscopic nipple-sparing subcutaneous mastectomy combined with immediate reconstruction using prosthesis implantation (SIE-NSM-IRPI) with those of conventional open-nipple and areola-sparing subcutaneous mastectomy combined with immediate reconstruction using prosthesis implantation (C-NSM-IRPI).

**Methods:**

In this retrospective cohort study, 64 early-stage breast cancer patients were divided into SIE-NSM-IRPI (n = 38) and C-NSM-IRPI (n = 26) groups. Perioperative results (operation time, intraoperative blood loss, incision length, drainage duration, and recent complications) were then compared between the two groups. Differences in satisfaction with the breasts, psychosocial well-being, physical well-being (chest) and sexual well-being were analyzed according to the BREAST-Q scale, and survival outcomes were also compared.

**Results:**

The median follow-up time was 51.5 months. The incision length of SIE-NSM-IRPI was shorter than that of C-NSM-IRPI (*P* < 0.001). SIE-NSM-IRPI achieved the same detection rate and median number of sentinel lymph nodes as C-NSM-IRPI (3.00vs. 4.00, *P* = 0.780). The incidence of prosthesis removal due to infection or prosthesis exposure in the SIE-NSM-IRPI group was lower than that in the C-NSM-IRPI group (*P* = 0.015). Satisfaction with breasts (82.00vs.59.00, *P* < 0.001), psychosocial well-being (93.00vs.77.00, *P* = 0.001) and physical well-being (chest) (89.00vs.82.00, *P* < 0.001) scores were higher in the SIE-NSM-IRPI group. There were no significant differences between the two groups in disease-free survival (hazard ratio = 0.829, 95% confidence interval = 0.182–3.779) and overall survival (hazard ratio = 1.919, 95% confidence interval = 0.169–21.842).

**Conclusion:**

In this selected cohort of patients with early breast cancer, SIE-NSM-IRPI was comparable to C-NSM-IRPI, considering oncologic safety and detection of sentinel lymph nodes. It had a lower incidence of prosthesis removal, shorter incision length, and was associated with better patient satisfaction with the breasts. More random clinical trials of this novel approach in a larger cohort of Chinese patients with an extended follow-up period are needed in the future.

**Supplementary Information:**

The online version contains supplementary material available at 10.1007/s00464-023-09862-6.

Breast cancer is the most common malignant tumor in female patients [1]. The systematic treatment of breast cancer has dramatically improved in recent years. While radical resection surgery can achieve a curative effect, there is increased research focus on achieving an improved breasts appearance and quality of life for patients. Nipple-sparing subcutaneous mastectomy (NSM) combined with immediate prosthetic reconstruction (IPR) has become one of the most common breast surgeries for breast cancer [2]. Conventional open NSM-IPR(C-SIE-NSM-IPR) leaves noticeable surgical scars on the breast surface. Notably, the incision is under the tension of the prosthesis, resulting in the incision splitting, increasing the risk of prosthetic exposure [3]. Some surgeon give incision incise around the areola to accomplish NSM, however, this could result in increased necrosis of the nipple-areola complex [4, 5]. Therefore, we designed a new procedure: single-port insufflation endoscopic nipple-sparing mastectomy with immediate prosthetic reconstruction (SIE-NSM-IPR). A small single-port incision on the lateral aspect of the chest wall is used to perform resection of the gland and implant the prosthesis simultaneously, minimizing the surgical scar and ensuring that it is not in a high-tension area. This strategy also improves the postoperative appearance and effectively reduces surgical complications. Cases of early breast cancer treated at Beijing Friendship Hospital affiliated with Capital Medical University were compared to explore differences in the efficacy of SIE-NSM-IPR and C-NSM-IPR.

## Methods

### Patients and analysis

Before the surgery, doctors fully communicated with the patients through the strategy of “sharing decision-making”. The patients chose the surgical techniques. Patients provided written informed consent for the publication of this retrospective cohort study when they came to the outpatient clinic for re-examinations.

The inclusion criteria were as follows: (a) age between 18 and 70 years; (b) diagnosis of stage I or II invasive breast carcinoma or carcinoma in situ; (c) original lesion ≤ 3 cm in diameter; (d) distance between the lesion and the nipple-areola complex ≥ 2 cm; (e) clinically negative axillary nodes; (f) tumor constrained to the mammary gland; (g) tumor is not invading the nipple-areola complex or the skin; (h) negative sentinel lymph node biopsy; (i) Eastern Cooperative Oncology Group score of 0–2; (j) patient not suitable for breast-conserving surgery and requesting breast reconstruction.

Patients with a positive sentinel lymph node biopsy were excluded from the study as they should undergo axillary lymph node dissection or radiotherapy. All patients underwent surgery for breast cancer (*n* = 64) between January 2014 and December 2019 at Beijing Friendship Hospital affiliated with Capital Medical University. The patients were divided into two groups: SIE-NSM-IRPI (*n* = 38) and C-NSM-IRPI (*n* = 26) (Table 1). The length of the incision, cosmetic score, procedure time, intraoperative blood loss, postoperative drainage time, total lacuna drainage, complications and survival outcome data of the two groups were compared.

**Table 1:** Patient characteristics (*n* = 64)

	SIE-NSM–IPR group (*n* = 38)	C-NSM-IPR group (*n* = 26)	*P*-value
Age(years)			0.129
Median(25,75percentile, range)	42.00 (36.75, 51.75, 42.00)	45.50 (39.00, 59.00, 42.00)	
Range	28–64	28–70	
BMI(kg/m2)			0.006
Median(25,75percentile, range)	21.91 ( 19.98, 24.10, 14.14)	25.57 (21.11, 28.10, 12.07)	
Tumor location, n(%)			0.872
Lateral-upper quadrant	18 (47.4%)	13 (50.0%)	
Lateral-lower quadrant	9 (23.7%)	4 (15.4%)	
Medial-upper quadrant	6 (15.8%)	5 (19.2%)	
Medial-lower quadrant	5 (13.2%)	4 (15.4%)	
The distance between tumor and nipple-areola complex (cm)			0.190
Median(25,75percentile, range)	3.00 (2.00, 4.00, 4.00)	2.00 (2.00, 4.60, 7.50)	
Neoadjuvant chemotherapy			> 0.999
Yes	5 (13.2%)	3 (11.5%)	
No	33 (86.8%)	23 (88.5%)	
T stage			0.010
Situ	10 (26.3%)	4 (15.4%)	
T1im	0 (0%)	3 (11.5%)	
T1a	1 (2.6%)	5 (19.2%)	
T1b	6 (15.8%)	4 (15.4%)	
T1c	15 (39.5%)	3 (11.5%)	
T2	6 (15.8%)	7 (26.9%)	
Degree of invasive carcinoma differentiation, *n* (%)			0.614
Situ or deficiency	14 (38.9%)	8 (30.8%)	
G1	5 (13.8%)	3 (11.5%)	
G2	12 (33.3%)	13 (50.0%)	
G3	5 (13.8%)	2 (7.7%)	
ER, n(%)			0.746
Positive	32 (84.2%)	21 (80.8%)	
Negative	6 (15.8%)	5 (19.2%)	
PgR, n(%)			0.191
Positive	25 (65.8%)	21 (80.8%)	
Negative	13 (34.2%)	5 (19.2%)	
HER2, n(%)			0.935
Positive	7 (18.4%)	5 (19.2%)	
Negative	31(81.6%)	21 (80.8%)	
Tumor thrombus			0.062
Positive	0(0%)	3 (11.5%)	
Negative	38 (100%)	23 (88.5%)	

BMI body mass index; ER Estrogen Receptor; PgR Progesterone Receptor; HER2, human epidermal growth factor receptor, type 2. HER2 status was estimated by immunohistochemistry or in situ hybridization (IHC or FISH). Tumors were considered to be HER2 positive if the average IHC showed (+ + +), HER2 gene/chromosome 17 ratio was ≥ 2 or the average HER2 gene copy number was ≥ 6

### Surgical procedures

**Fig. 1:**
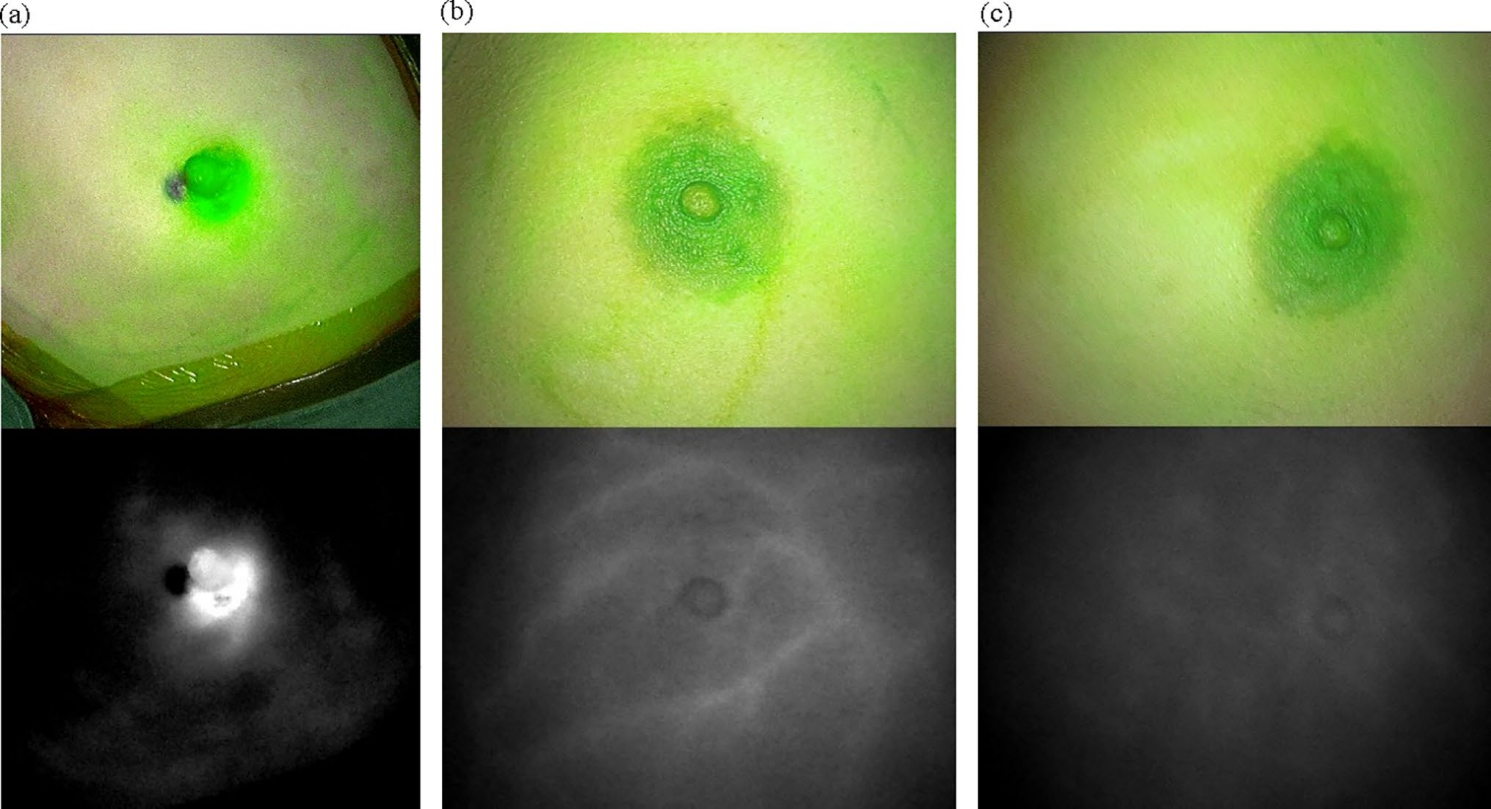
Blood supply mode of the nipple-areola complex **(a** Type V1,** b** Type V2 and **c** Type V3)

All surgeries were performed by a single surgeon (Dr. Xiang Qu), who is well-experienced in breast cancer surgery and familiar with breast-endoscopic surgery. The patients were in a supine position, with the ipsilateral arm abducted at 90°. To classify the blood supply mode of the nipple-areola complex, 4 ml of 2.5 mg/ml Indocyanine Green Solution (25 mg; Yichuang Pharmaceutical LLC, Dandong, China) was injected by intravenous drip. We performed the Indocyanine Green angiography after confirming that the tests for iodic allergy were negative before the surgery. The blood supply to the nipple-areola complex is classified according to where the perfusion originates [6–9] and was classified as types V1, V2 and V3 (Fig. 1); using a Fluorescence Imaging System (Optomedic Technique Inc., Guangdong, China). Type V1 originates predominantly from the underlying breast tissue, type V2 originates from the surrounding skin, and type V3 is a combination of V1 and V2. Type V1 indicates that the nipple–areola complex blood supply mainly comes from the glands in a vertical direction, increasing the probability of nipple-areola complex ischaemic necrosis after NSM. Soft-tissue expander replacement prosthesis implantation was chosen to reduce the pressure of the implant on the nipple–areola complex. For types V2 or V3, the surgical procedures were performed as described.

### The SIE-NSM-IPR group

**Fig. 2:**
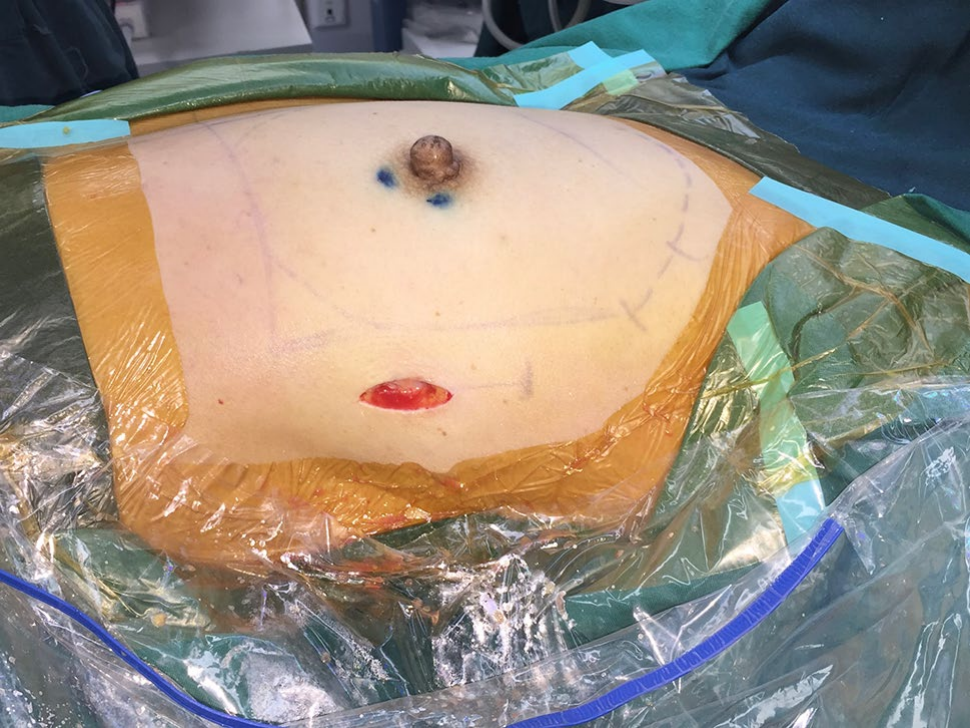
A 2.5 cm single-port small incision was performed on the side chest wall at the axillary front-flat nipple level

**Fig. 3:**
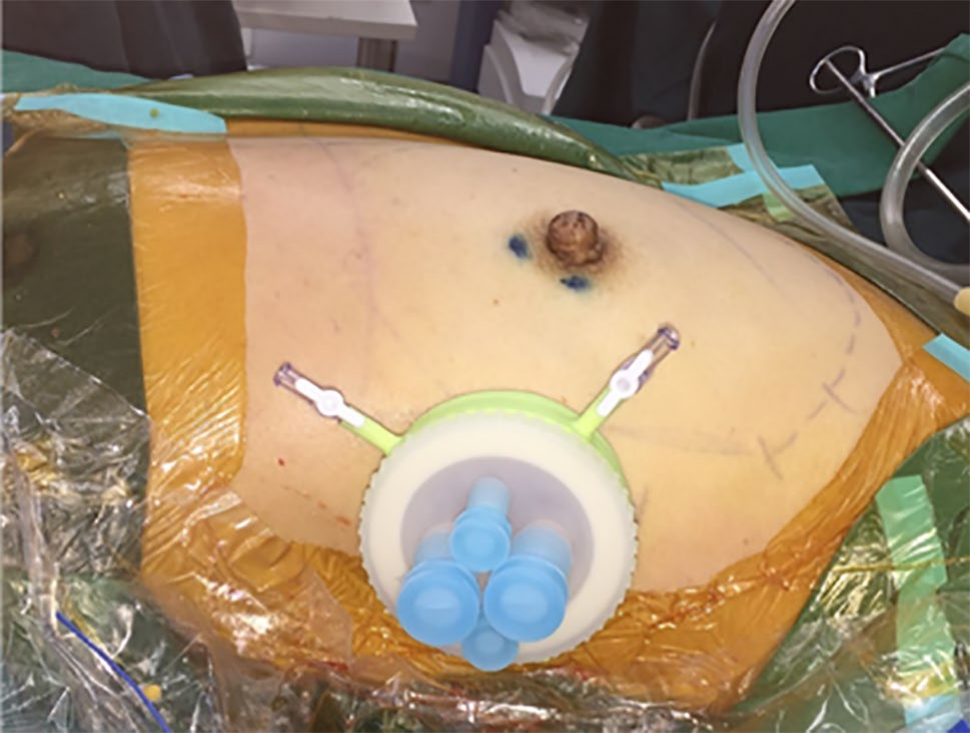
Single-port insufflation kit was placed through the single-port incision

The optics, trocars and endoscopic instruments were all provided in a laparoscopy equipment kit and were reusable (Olympus Optical Co., Tokyo, Japan). A single-port small incision was performed on the side chest wall at the axillary front-flat nipple level to complete gland resection and prosthesis implantation at the same time (Fig. 2). To create space for the endoscopic surgical procedure, tumescent solution (1 mg of adrenaline and 20 ml of 2% lidocaine in a mixture of 250 ml 0.9% sodium chloride and 250 ml sterilized distilled water) was used to infiltrate the axillary fat pad (250–500 ml) and the fat overlying the pectoralis major (1000–1500 ml) through the single-port incision. After 10–15 min, aspiration of the adipose tissue was initiated at a pressure of 800 mbar, with the liposuction cannula inserted through the incision. Liposuction was conducted in the retromammary space to separate the gland and the pectoralis major. When the liquid sucked out was light pink, the liposuction ended. The single-port insufflation kit (HTKD-Hang T Port, China) was placed through the single-port incision (Fig. 3). An adequate working space was created by insufflation with carbon dioxide gas (8 mmHg) to perform the sentinel lymph node biopsy before NSM. With magnification by the endoscope, it was easy to distinguish the sentinel lymph nodes stained with a sentinel lymph node tracer (Nano-Carbon). The sentinel lymph nodes were separated, and the surrounding connective tissue and lymphatic vessels were cut to complete the sentinel lymph node biopsy. Fast-frozen sections of the sentinel lymph nodes were evaluated intraoperatively. If metastases were present in the sentinel lymph nodes, the patient underwent axillary lymph node dissection or radiotherapy, and soft tissue expander implantation was performed instead of immediate prosthetic reconstruction.

**Fig. 4:**
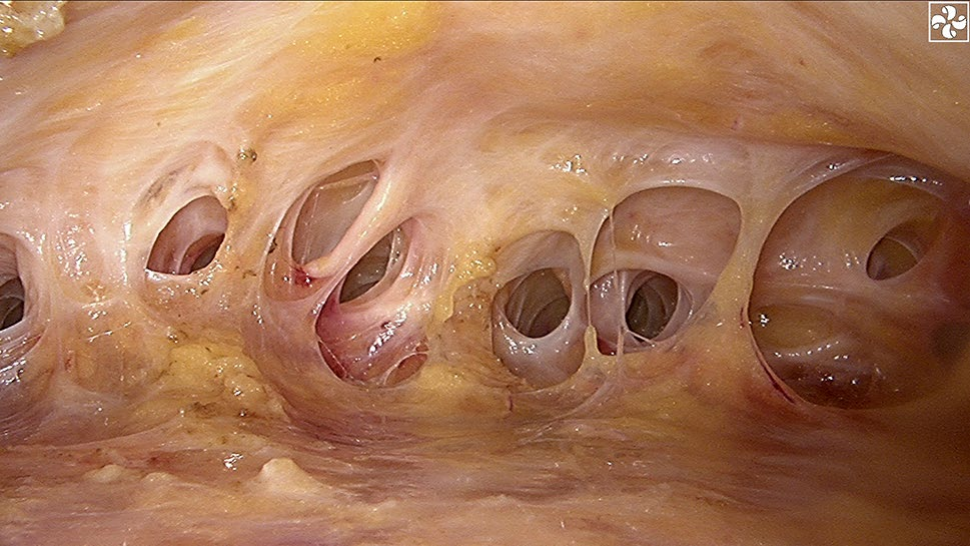
The retromammary space

**Fig. 5:**
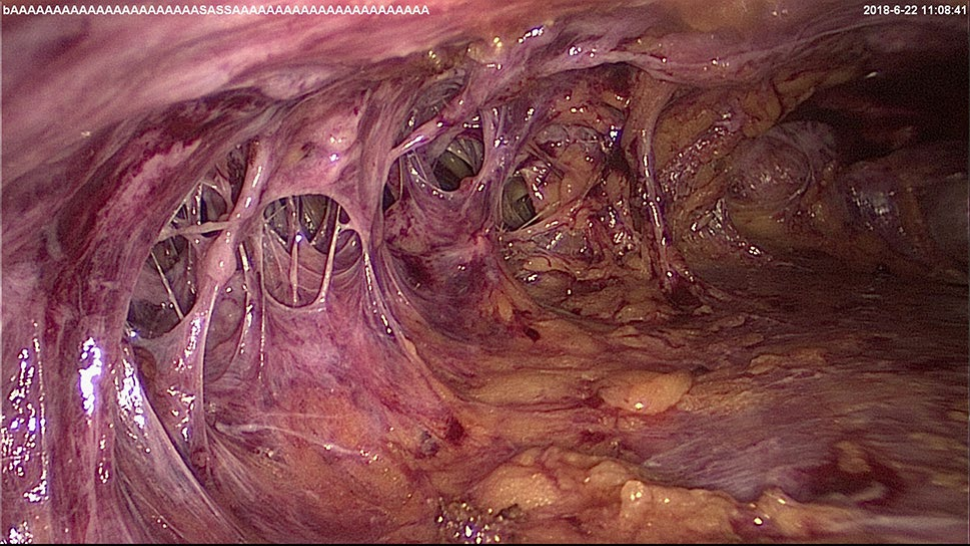
Triangular bundle ligaments

**Fig. 6:**
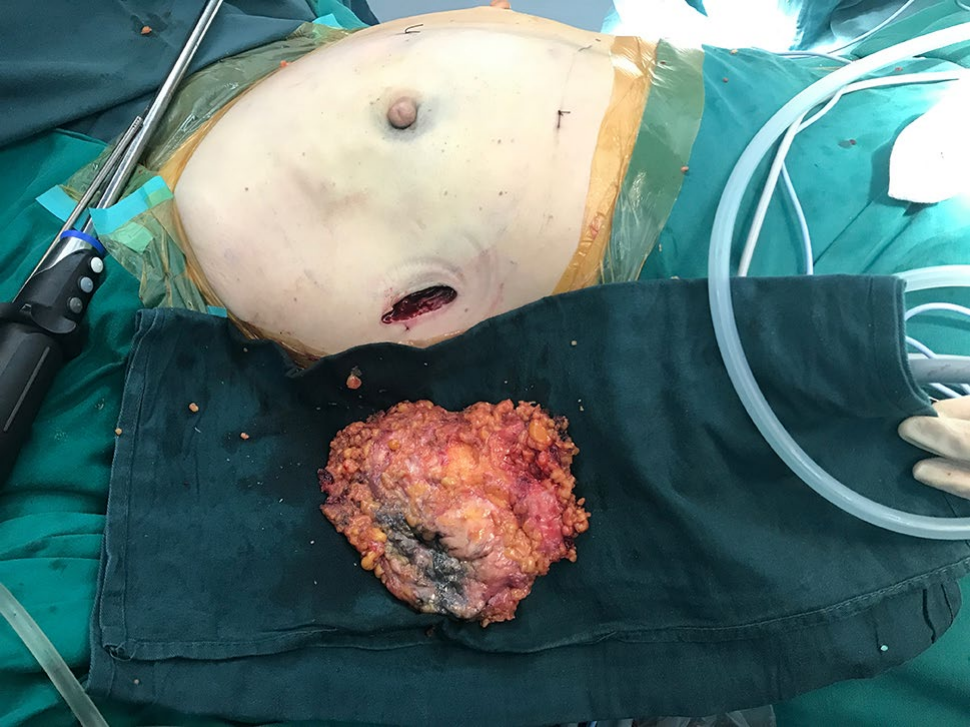
The entire breast gland was removed through the base of the single-port insufflation kit

Then, SIE-NSM was performed through the single-port insufflation kit. The retromammary space was first separated after liposuction and carbon dioxide input (Fig. 4). The mammary gland and pectoralis major were easily separated by cutting the fibrous connective tissue. The mammary gland was then separated from the skin flap by severing Cooper’s ligaments with a scalpel. Following this, the gland was connected to the body by only peripheral ligaments, including clavicular ligaments, medial sternal ligaments, lateral pectoralis major ligaments, part of the horizontal ligaments and triangle bundle ligaments (Fig. 5). The gland was completely dissociated after breaking the ligaments with an endoscopic electrotome. To be specific, the extent of dissection was from the subclavian to the submammary fold and from the parastern to the anterior axillary line. The entire breast gland was then removed through the base of the single-port insufflation kit to prevent incision metastases (Fig. 6) and the resected glandular tissue was sent routinely for histopathology. The stump of the nipple site and the surface of the tumor were intraoperatively submitted to a fast-frozen pathological test to determine if there was tumor invasion. If tumor invasion was present, the nipple-areola complex and the skin on the tumor were dissected.

**Fig. 7:**
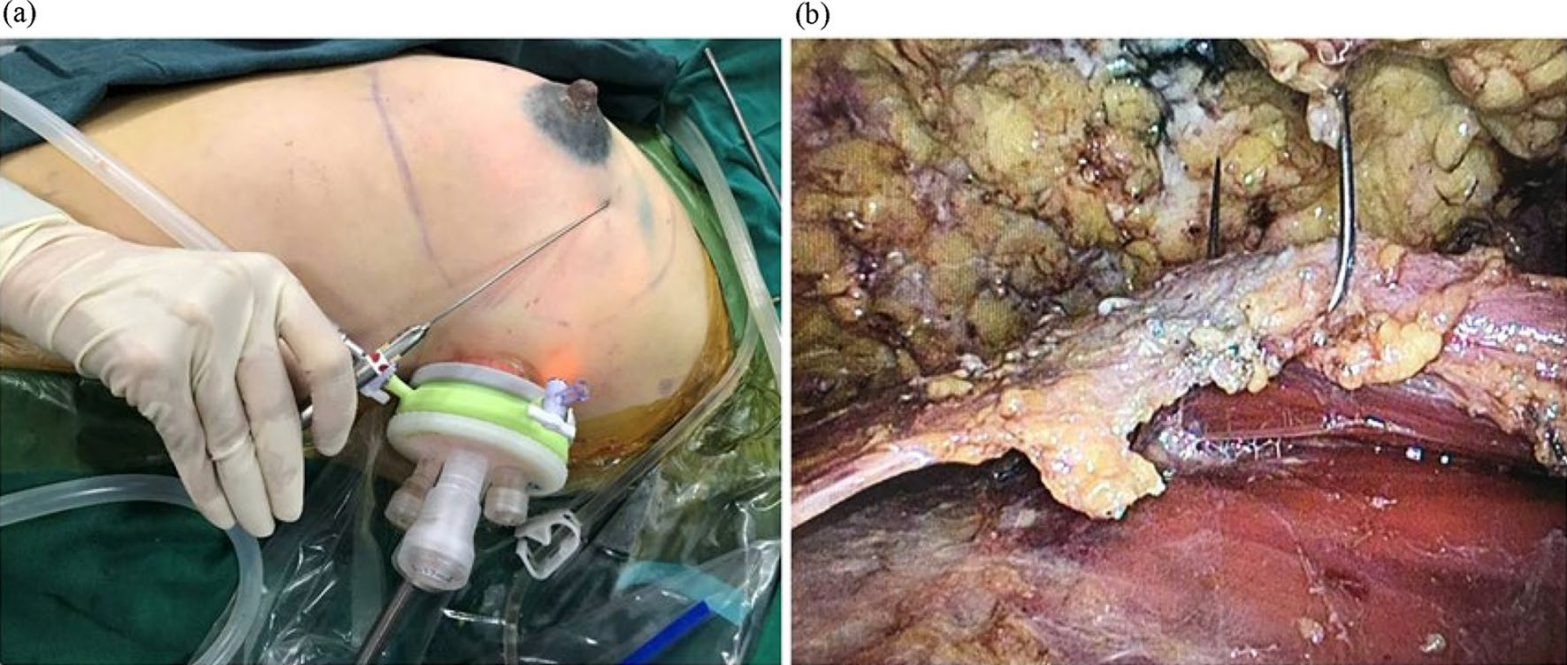
**a** The special retractor was passed through the skin of the chest wall. **b** The retractor was used to suspend the pectoralis major muscle in vitro

**Fig. 8:**
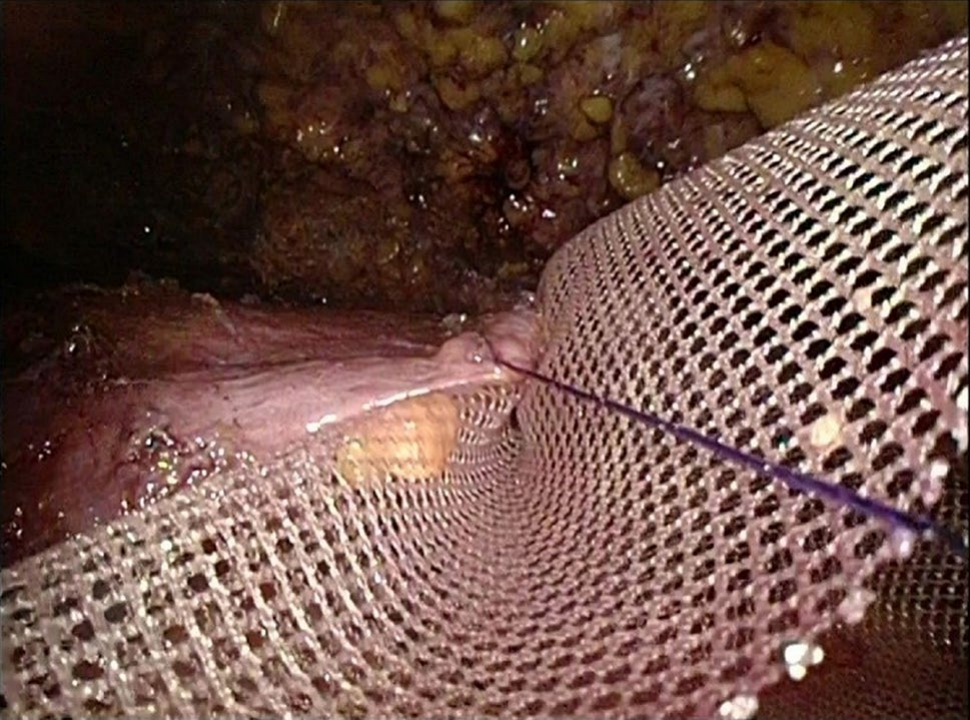
The lower edge of the pectoralis major were sutured continuously with a 2–0 barbed suture under the endoscope

**Fig. 9:**
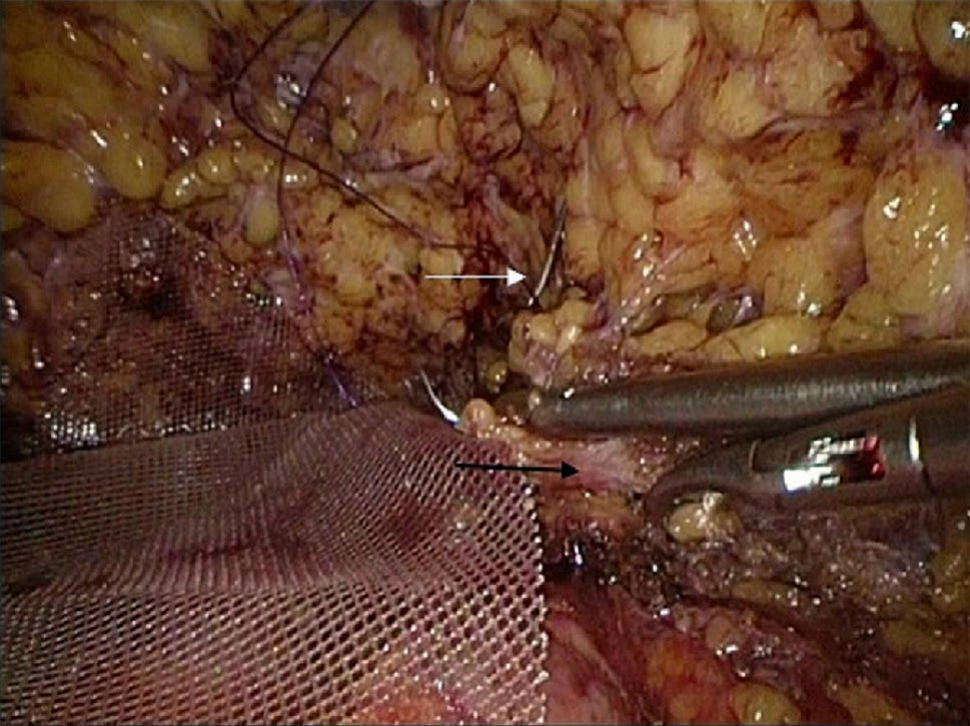
The other end of the TiLOOP and the remaining triangular bundle ligaments were sutured together, thus forming a pouch. The white arrow marked the triangular bundle ligaments and the black arrow marked the needle positioning the submammary wrinkle

**Fig. 10:**
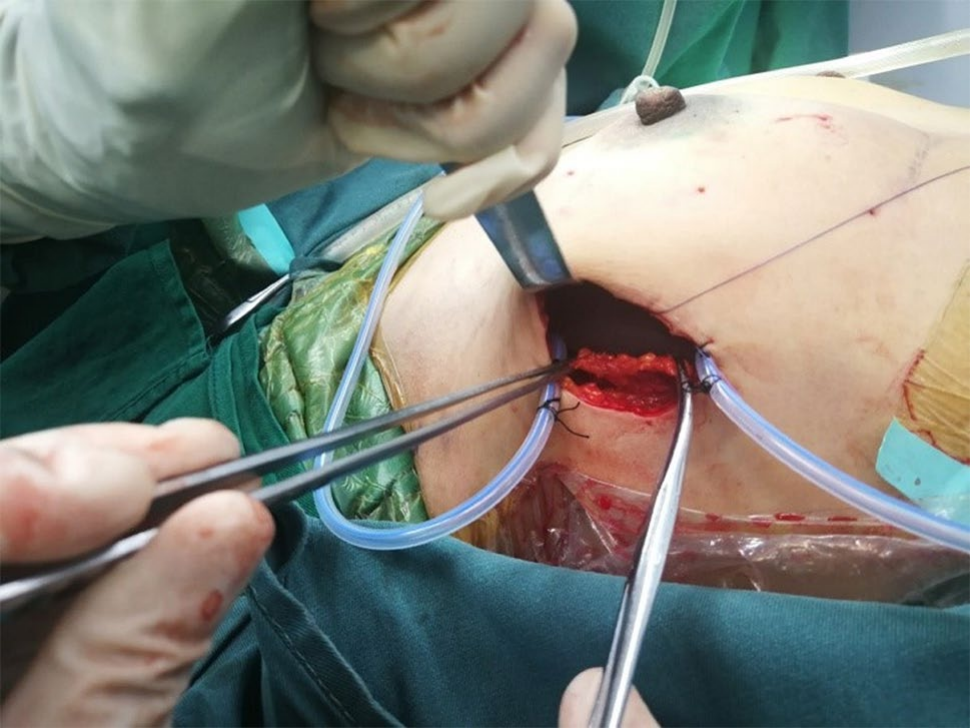
The residual lateral fusion fascia of the pectoralis major muscle was sutured through the single-port incision to close the capsule

The deep fascia’s superficial layer covering the major and minor pectoralis muscle space was cut with an ultrasonic knife. A special retractor was passed through the skin of the chest wall and used to suspend the pectoralis major muscle in vitro (Fig. 7) to expose the pectoralis major muscle space better. The starting point of the pectoralis major was dissected with an ultrasonic knife, and the pectoralis muscle space was separated to the lateral side of the anterior midline by 1.5 cm. One end of the TiLOOP artificial patch material (PFM medical titanium GmbH, Germany) and the lower edge of the pectoralis major were sutured continuously with a 2–0 barbed suture under the endoscope (Fig. 8). The other end of the TiLOOP and the remaining triangular bundle ligaments were sutured together, thus forming a pouch (Fig. 9) to provide sufficient support for the prosthesis. After flushing the space, the prosthesis was implanted through the single-port incision. The operating table was adjusted to approximately a 60° sitting position and the patient’s bilateral symmetry was compared. One silicone drainage tube was indwelled on the cephalic side, and the other was indwelled on the pouch’s foot side. Another silicone drainage tube was indwelled cephalically on the head side of the superficial layer of the pouch, coming out from a new hole. The drainage tube was connected to negative pressure suction. The residual lateral fusion fascia of the pectoralis major muscle was sutured through the single-port incision to close the capsule (Fig. 10). The single-port incision was closed by intermittent suture for subcutaneous tissue and intradermal continuous suture with absorbable sutures.

### The C-NSM-IPR group

The operative position and the sentinel lymph node tracing were the same as described above for SIE-NSM-IPR. An external quadrant radial incision was made on the surface of the breast, approximately 12 cm in length. Aside from preserving all the skin and the nipple-areola complex, the scope and principle of the surgical procedures were the same as for open mastectomy and sentinel lymph node biopsy. The subsequent procedures such as forming the pouch and implanting the prosthesis were similar to SIE-NSM-IPR but through an open incision.

### Postoperative management

The drainage tube was removed when the drainage volume was less than 30 ml for 3 consecutive days. The patients wore a compression bra for 2 weeks after the drainage tube removal. All patients accepted the necessary adjuvant therapy according to the National Comprehensive Cancer Network guidelines if necessary.

### Cosmetic evaluation, patient satisfaction and follow-up

The patients’ aesthetic satisfaction was evaluated by the BREAST-Q [10] 6 months after surgery. The BREAST-Q includes questions about satisfaction with breasts, psychosocial well-being, physical well-being (chest) and sexual well-being. The scores were compared between the two groups. All patients were followed up at the outpatient clinic for re-examination or through a telephone call every 6 months to record whether there was local recurrence or metastasis.

### Statistical analysis

Continuous variables with normal distribution were reported as mean values (standard deviation [SD]). Non-normal variables were presented as medians (interquartile range [IQR]). The means of two continuous normally distributed variables were compared using independent samples Student’s t-test. Mann–Whitney U test was used to compare the means of two groups of variables not normally distributed.Log-rank tests were used to compare the overall survival (OS) and disease-free survival (DFS) rates between the groups. A *p-*value less than 0.05 was considered statistically significant. All reported *p *values were two-sided. The statistical analyses were performed using SPSS v24 (IBM Corp., Chicago, IL, USA).

## Results

Considering that the body mass index (BMI) and T stage were different between the two groups (Table 1), the Spearman test was undertaken to investigate whether the bias affected the results of quality of life and post operative complications. The result indicated that the correlation between BMI/T stage and post operative complications was not statistically significant. Although a correlation existed in some aspects of the quality of life, the correlation coefficient was weak (see Supplementary Table I). The baseline bias might be acceptable.The mean incision length was 3.66 ± 1.05 cm in the SIE-NSM-IPR group and 11.12 ± 3.29 cm in the C-NSM-IPR group. The difference between the two groups was statistically significant (*P* < 0.001) (Table 2). In the SIE-NSM-IPR group, the mean operative time was 300.97 ± 64.77 min, which was significantly longer than that in the C-NSM-IPR group (196.88 ± 59.79 min) (*P* < 0.001). There were no significant differences between the groups regarding intraoperative blood loss, sentinel lymph nodes detection rate, number of sentinel lymph nodes, drainage duration, and total drainage volumes. However, the prosthesis exposure or incision disruption rate was significantly lower in the SIE-NSM-IPR group, at 2.6% vs. 23.1% (*P* = 0.015). Notably, there was a lower trend in the rate of necrosis of the nipple-areola of the SIE-NSM-IPR group than in the C-NSM-IPR group.

**Table 2:** Comparison of perioperative results based on surgical procedure

Variable	SIE-NSM-IPR group (*n* = 38)	C-NSM-IPR group (*n* = 26)	*P*-value
Operation time (min)			< 0.001
Mean ± SD	300.97 ± 64.77	196.88 ± 59.79	
Intraoperative blood loss (ml)			0.073
Median(25,75percentile, range)	30.00 (20.00, 50.00, 90.00)	20.00 (20.00, 35.00, 92.00)	
Incision length (cm)			< 0.001
Mean	3.66 ± 1.05	11.12 ± 3.29	
Range	2.5 -8	5 -15	
Detection rate of Sentinel lymph nodes			> 0.999
	100%	100%	
Sentinel lymph nodes, n			0.780
Median(25,75percentile, range)	3.00 (2.75, 4.25, 5.00)	4.00 (2.00, 5.25, 5.00)	
Drainage duration (day)			0.515
Median(25,75percentile, range)	8.50 (8.00, 10.50, 9.00)	9.00 (7.75, 12.00, 14.00)	
Total drainage volume (ml)			0.042
Mean ± SD	645.16 ± 224.99	772.96 ± 263.83	
Necrosis of nipple-areola, n (%)	2 (5.3%)	6 (23.1%)	0.054
Prosthesis exposure or incision disruption, n (%)	1 (2.6%)	6 (23.1%)	0.015
Infection, n (%)	1 (2.6%)	4 (15.4%)	0.149
Local recurrence, n (%)	1 (2.6%)	1 (3.8%)	> 0.999
Metastasis, n (%)	4 (10.5%)	3 (11.5%)	> 0.999

Six months after surgery, the patients completed the BREAST-Q scale. The median (25, 75 percentile, range) scores of satisfaction with breasts (*P* < 0.001), psychological well-being (*P* = 0.004), and physical well-being (chest) (*P* < 0.001) were statistically different between the SIE-NSM-IPR group and C-NSM-IPR group. There was no statistically significant difference in sexual well-being (*P* = 0.052) between the two groups (Table 3).

**Table 3:** Breast-Q scale scores of patients

	SIE-NSM-IPR group (*n* = 38) Median (25,75percentile,range)	C-NSM-IPR group (*n* = 26) Median (25,75percentile,range)	*P*-value
Satisfaction with breasts	82.00 (75.00,91.00,45.00)	59.00 (59.00,76.75,41.00)	< 0.001
Psychological well-being	93.00 (83.00,100.00,57.00)	77.00 (77.00,84.00,36.00)	0.001
Physical well-being (chest)	89.00 (82.00,100.00,18.00)	82.00 (77.00,83.75,41.00)	< 0.001
Sexual well-being	84.00 (69.00,85.75,61.00)	66.00 (66.00,84.00,52.00)	0.052

BREAST-Q® VERSION 2.0^©^ Memorial Sloan Kettering Cancer Center and The University of British Columbia, 2017

All patients were followed up. One patient (2.6%) in the SIE-NSM-IPR group experienced local recurrence, four patients (10.5%) experienced metastasis, and two patients (5.3%) died. One of these patients died from respiratory failure caused by lung metastasis, and the other died from respiratory and liver failure caused by lung and liver metastases. One patient (3.8%) in the C-NSM-IPR group experienced local recurrence and three patients (11.5%) experienced metastases; one patient (3.8%) died from respiratory failure caused by lung metastasis. There were no significant differences between the two groups in the DFS (hazard ratio = 0.829, 95% confidence interval = 0.182–3.779) and OS (hazard ratio = 1.919, 95% confidence interval = 0.169–21.842) (Table 4).

**Table 4:** Differences in survival between the two groups

	OS			DFS		
	HR	95%CI		HR	95%CI	
C-NSM-IPR group	Ref			Ref		
SIE-NSM-IPR group	1.919	0.169–21.842		0.829	0.182–3.779	

## Discussion

Nipple-sparing subcutaneous mastectomy combined with immediate breast reconstruction with prosthesis implantation is the best choice for patients with high breast appearance requirements and those who are unsuitable for breast-conserving surgery [11, 12]. There are several problems with traditional open reconstruction. First, regardless of whether the incision is performed on the breast surface or lateral chest wall, it will leave an obvious surgical scar. Moreover, incisions on the breast surface bear the tension of the prosthesis, which can cause scar widening, incision disruption, prosthesis exposure and implant loss [13, 14]. To address these issues we designed SIE-NSM-IPR. This shortens the incision length and reduces the number of incisions as sentinel lymph node biopsy, NSM and reconstruction are performed through a single incision. In addition, by designing the incision in the axillary midline, the natural sagging of the upper limb and corset can cover the postoperative scar. The incision tension caused by the prosthesis can be effectively moderated, reducing the risk of severe complications such as wound-disruption and implant removal.

This study compares the oncologic safety, perioperative results and aesthetic outcomes of SIE-NSM-IRPI and C-NSM-IRPI. There were no significant differences in age, tumor size, molecular subtype, intraoperative blood loss, detection rate of sentinel lymph nodes and number of sentinel lymph nodes between the groups. The incision length in the SIE-NSM-IRPI group was significantly shorter than that in the C-NSM-IRPI group. Moreover, the membrane anatomy and ligaments around the mammary gland were clear under the magnification of the endoscope. It was necessary to cut these ligaments without destroying the membrane’s anatomy [15], reducing heat injury and fat liquefaction. Due to liposuction in SIE-NSM surgery, the fat content in the breast is considerably reduced, thus reducing the occurrence of fat liquefaction.

In the SIE-NSM-IRPI group, two cases (5.3%) experienced necrosis of the nipple-areola. In one case, the patient’s nipples fell off spontaneously and experienced nigrescence after ischemia; however, the skin healed well. The second case experienced infection and prosthesis exposure due to necrosis of the nipple-areola, resulting in the removal of the prosthesis followed by capsule excision and incision suturing. The patient healed well after the surgery. In the C-NSM-IRPI group, six cases (23.1%) experienced necrosis of the nipple-areola. One case suffered from necrosis of the areola and infection of the operation region, and her nipple also fell off. After antibiotic therapy, the infection resolved, and the prosthesis was not removed. The prostheses in the other cases were removed due to infection. Necrosis of the nipple–areola is a common complication of NSM and breast reconstruction [5, 16–20]. It is associated with capillary network injury in the nipple-areola region, excessive epidermal tension and insufficient blood supply induced by prosthesis implantation. The nipple–areola complex ischemia or necrosis rate was low in the SIE-NSM-IRPI group, mainly because the single-port incision was hidden in the armpit. This avoided a breast surface or areolar incision and further mitigated direct damage to the blood supply in the nipple-areolar region.

In the later stages of breast surgeries, the blood supply pattern and necrosis risk of the nipple-areola complex are evaluated using Indocyanine Green fluorescence angiography technology. In this study, type V2 and type V3 patients were identified in the groups. For type V1 cases, prothesis implantation was via a soft tissue expander to relieve the influence of pressure on the blood supply of the nipple–areola complex.

The rate of prosthesis exposure or incision disruption in the SIE-NSM-IRPI group was lower than that in the C-NSM-IRPI group, because the incision was transferred to the lateral chest wall. This avoided the incision tension brought about by prosthesis implantation and lowered the risks of incision splitting and prosthesis exposure.

Designed and proposed by Pusic et al., the BREAST-Q scale includes satisfaction with breasts, psychological well-being, physical well-being (chest), and sexual well-being. It has good reliability and validity when used to evaluate the quality of breast reconstruction [21]. The endoscopic surgery group patients obtained high BREAST-Q scores, indicating that by avoiding a breast surface incision, this surgical method was associated with enhanced patient satisfaction with the reconstructed breasts, improved postoperative chest wall status, and better psychological well-being outcomes.

At follow-up, no statistically significant differences were observed between the two groups regarding DFS and OS, demonstrating that SIE-NSM-IPR is safe and effective. Although the endoscopic surgery time was longer than that for traditional open surgery, the anatomical structure were more visible due to the fat dissolution and liposuction steps, and the anatomical positioning in each step was more precise due to magnification provided by the endoscope. Additionally, the incidence of complications was reduced by protecting the essential structures. A future prospective comparative study is planned to further verify the safety and feasibility of this surgical method through more extended follow-up support of patients.

## Conclusion

In this selected cohort of patients with early breast cancer, SIE-NSM-IRPI was comparable to C-NSM-IRPI in terms of oncologic safety and detection of sentinel lymph nodes. It had a lower incidence of prosthesis removal, and a shorter incision length, and was associated with better patient satisfaction with the breasts. Since this was a retrospective study and patients were only followed up for a short duration, we have designed a random clinical trial to evaluate the medical and cosmetic outcomes of this novel approach in a larger cohort of Chinese patients with an extended follow-up period.

## Supplementary Information

Below is the link to the electronic supplementary material.

Supplementary file1 (DOCX 15 kb)
