# Supplementary material for: Single-port nipple-sparing subcutaneous mastectomy with immediate prosthetic breast reconstruction for breast cancer
Source: Surg Endosc. 2023 Jan 25;37(5):3842–51. doi: 10.1007/s00464-023-09862-6 (PMC10156621; doi:10.1007/s00464-023-09862-6)
Supplement: Supplementary file 1 — Supplementary file1 (DOCX 15 kb) [file 464_2023_9862_MOESM1_ESM.docx]

[Supplementary](javascript:;)

Table I BMI and T-stage influence to the study findings

|  | BMI | | T | |
| --- | --- | --- | --- | --- |
|  | correlation coefficient | P value | correlation coefficient | P value |
| Satisfaction with the breasts | 0.264 | 0.035 | 0.131 | 0.303 |
| Psychosocial wellbeing | 0.312 | 0.012 | 0.139 | 0.273 |
| Physical wellbeing chest | 0.306 | 0.014 | 0.306 | 0.014 |
| Sexual wellbeing | 0.198 | 0.116 | 0.093 | 0.463 |
| Necrosis of nipple-areola | 0.102 | 0.421 | 0.085 | 0.504 |
| Prosthesis exposure or incision disruption | 0.005 | 0.966 | 0.080 | 0.527 |
| infection | 0.055 | 0.665 | 0.103 | 0.417 |

Table II Complications grades according to Clavien-Dindo

| Clavien-Dindo grade | SIE-NSM-IRPI | C-NSM-IRPI |
| --- | --- | --- |
| I | 1 | 1 |
| II | 0 | 3 |
| IIIa | 0 | 0 |
| IIIb | 1 | 4 |
| IV | 0 | 0 |
| V | 0 | 0 |
